# Transdiagnostic Patterns of Grip Strength in Schizophrenia, Current Depression, and Remitted Depression

**DOI:** 10.1001/jamapsychiatry.2026.0144

**Published:** 2026-03-18

**Authors:** Sofie von Känel, Anastasia Pavlidou, Niluja Nadesalingam, Victoria Chapellier, Melanie G. Nuoffer, Lydia Maderthaner, Alexandra Kyrou, Alexios Malifatouratzis, Florian Wüthrich, Stephanie Lefebvre, Victor Pokorny, Zachary Anderson, Stewart A. Shankman, Vijay A. Mittal, Sebastian Walther

**Affiliations:** 1 Translational Research Center, University Hospital of Psychiatry and Psychotherapy, University of Bern, Bern, Switzerland; 2 Graduate School for Health Science, University of Bern, Bern, Switzerland; 3 Department of Research in School and Instruction, Institute of Educational Science, University of Bern, Bern, Switzerland; 4 Department of Basic and Clinical Neuroscience, Institute of Psychiatry, Psychology and Neuroscience, King’s College London, London, United Kingdom; 5 University Hospital of Old Age Psychiatry, University of Bern, Bern, Switzerland; 6 Department of Psychiatry, Psychosomatics, and Psychotherapy, Center for Mental Health, University Hospital of Würzburg, Würzburg, Germany; 7 Department of Psychology, Northwestern University, Evanston, Illinois; 8 Stephen M. Stahl Center for Psychiatric Neuroscience, Department of Psychiatry and Behavioral Sciences, Northwestern University, Chicago, Illinois

## Abstract

This study explores transdiagnostic patterns of grip strength in schizophrenia, current depression, and remitted depression.

Grip strength is a simple, reliable, and cost-effective indicator of physical and mental health and higher grip strength is associated with better cognitive functioning.^1^ Low grip strength is associated with mental disorders, such as schizophrenia and depression.^2^ To date, no study directly compared grip strength between these 2 disorders and its persistence beyond the active phases of depression. Additionally, associations between grip strength and clinical symptoms are scarce.

## Methods

We cross-sectionally examined grip strength in 533 participants pooled from 5 studies and 2 sites, including schizophrenia (n = 175), current depression (n = 79), remitted depression (n = 104), and healthy controls (n = 175). Diagnosis followed *DSM-5* criteria. Exclusions included traumatic brain injury, neurological disorders, and substance dependence (excluding nicotine) and any past or current mental disorder in healthy controls. In schizophrenia, negative symptoms were assessed with the Brief Negative Symptom Scale. Depressive symptoms were assessed with the Montgomery-Åsberg Depression Rating Scale for participants with current and remitted depression, and healthy controls. Grip strength was measured in kilograms by averaging 3 trials of the dominant hand using an electronic hand dynamometer (CAMRY, model EH101) for all participants. The study followed Strengthening the Reporting of Observational Studies in Epidemiology (STROBE) reporting guidelines. Prior to starting assessments, written informed consent was obtained from all participants. Study protocols adhered to the declaration of Helsinki and were approved by the local ethics committees.

Group differences in grip strength were tested with linear regression controlling for age and sex, followed by post hoc pairwise comparisons (Benjamini-Hochberg method). Associations with symptoms were examined using partial Spearman correlations with attention to potential sex-specific patterns. Multiple comparisons were adjusted with the Benjamini-Hochberg method (eMethods in Supplement 1). Analysis were performed in R Studio versions 2025.05.0+496 and 2024.12.1+563 (The R Project).

## Results

Group (*F*_3_ = 12.5; *P* < .001), age (*F*_1_ = 4.6; *P* < .05), and sex (*F*_1_ = 309.8; *P* < .001) were significant predictors of grip strength (Table; Figure, A), unlike inpatient status per sensitivity analysis. Post hoc comparisons revealed that grip strength was higher in healthy controls compared with schizophrenia (*t*_527_ = 2.4; *P* = .02), current (*t*_527_ = −4.9; *P* < .001), and remitted depression (*t*_527_ = 5.2; *P* < .001). Likewise, the schizophrenia group performed better than both the current (*t*_527_ = −2.9; *P* = .006) and remitted (*t*_527_ = −2.9; *P* = .006) depression groups, while no difference in grip strength was observed between both depression groups (*t*_527_ = −0.2; *P* = .80). Grip strength correlated negatively with overall negative symptoms in schizophrenia (ρ = −0.23; *P* = .01) (Figure, B), specifically with avolition, affect, and alogia domains (all ρ > −.17; *P* < .03) (Figure, C). Sex-specific analyses revealed associations between symptom severity and grip strength in males with schizophrenia (ρ = −0.28; *P* = .03) or depression (ρ = 0.48; *P* = .04), but not in females.

**Table.** Sample Characteristics

Characteristic	Mean (SD)			
	Schizophrenia (n = 175)	cMDD (n = 79)	rMDD (n = 104)	HC (n = 175)
Age, y	37.1 (12.6)	31.2 (11.0)	29.1 (9.4)	33.7 (12.0)
Sex, %				
Male	52.6	32.9	35.6	44.0
Female	47.4	67.1	64.4	56.0
Grip strength, kg	29.4 (10.6)	23.7 (9.8)	24.5 (10.5)	30.7 (12.1)
Male	34.8 (10.8)	32.1 (11.6)	33.8 (9.7)	40.4 (10.6)
Female	23.5 (6.6)	19.6 (5.0)	19.4 (6.7)	23.0 (6.5)
PANSS score total	69.5 (19.8)			
OLZ-eq, mg/d	14.3 (10.7)			
BNSS score total	34.1 (16.0)			
Avolition	5.8 (3.0)			
Anhedonia	8.6 (4.8)			
Social	5.4 (2.9)			
Affect	8.5 (4.9)			
Alogia	3.2 (3.4)			
*DSM-5* depression rating	0.877 (1.000)			
MADRS		27.7 (8.5)	5.9 (5.5)	2.0 (2.8)
Male		27.2 (9.0)	6.0 (5.5)	1.2 (2.0)
Female		28.0 (8.4)	5.8 (5.6)	2.4 (3.1)
MADRS item lack of drive		3.3 (1.5)	1.2 (1.3)	

Abbreviations: BNSS, Brief Negative Symptom Scale; cMDD, current major depressive disorder; HC, healthy controls; MADRS, Montgomery-Åsberg Depression Rating scale; PANSS, Positive and Negative Syndrome Scale; OLZ-eq, olanzapine equivalent; rMDD, remitted major depressive disorder.

**Figure.**
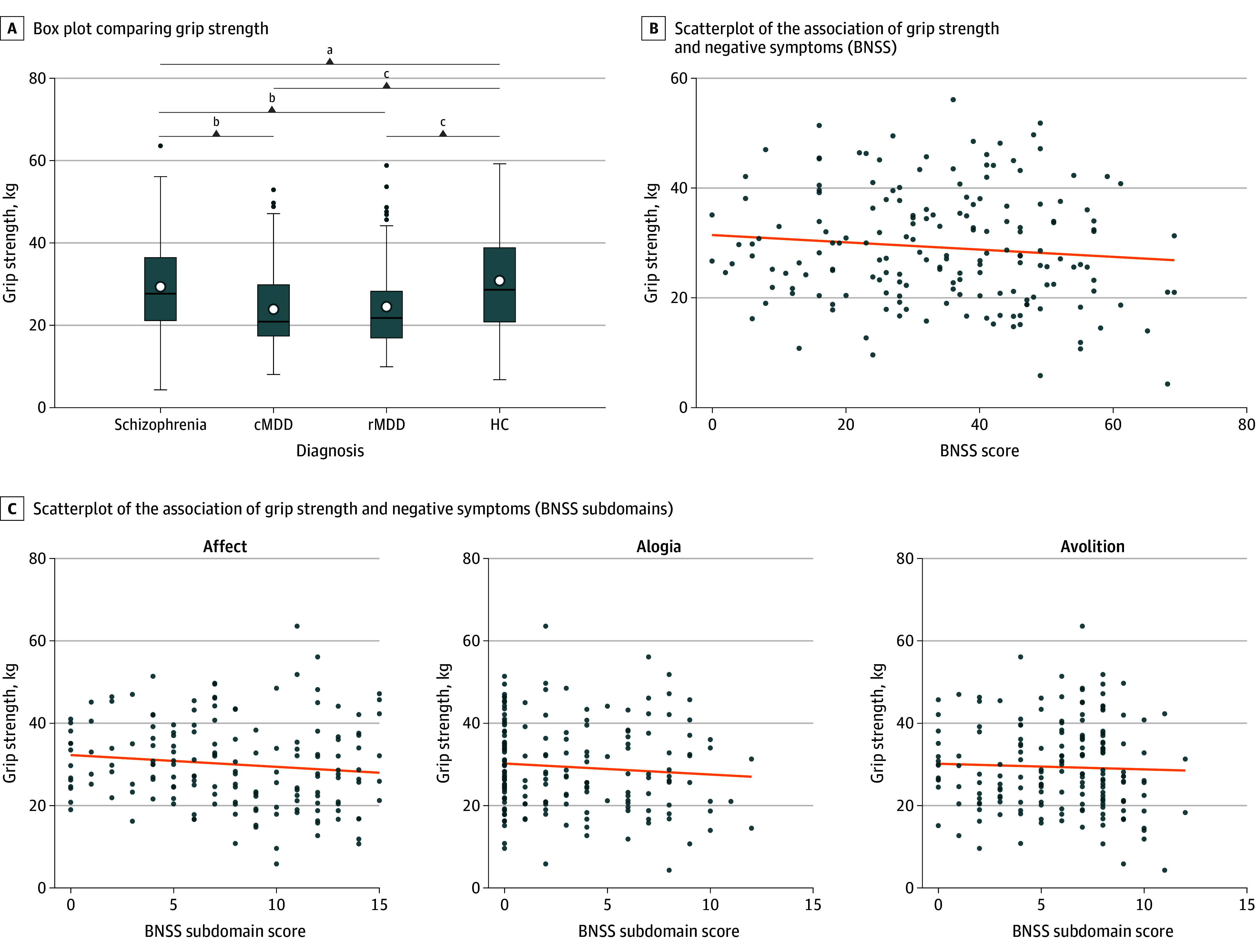
Box Plot and Scatterplots Comparing Group Differences BNSS indicates Brief Negative Symptom Scale; cMDD, current major depressive disorder; HC, healthy controls; rMDD, remitted major depressive disorder. ^a^*P* < .05. ^b^*P* < .01. ^c^*P* < .001.

## Discussion

This transdiagnostic study found reduced grip strength in schizophrenia, current depression , and remitted depression compared with healthy controls. Depression groups showed lower grip strength than schizophrenia, but with no difference between them.

These findings suggest that reduced grip strength reflects transdiagnostic mechanisms involving motor control, sensory integration, and motivation.^3,4^ In depression, persistent reductions during remission may reflect residual symptoms, such as psychomotor or executive dysfunction, indicating that remission does not fully normalize psychomotor performance.^5^ In schizophrenia, associations with negative symptoms, particularly in avolition, affect, and alogia domains, might highlight the role of disrupted frontal–striatal circuits and dopaminergic dysfunction in translating motivational drive into motor output.

The sex-specific pattern, with associations primarily in males, underscores the need to consider sex differences in both symptom expression and motor performance when interpreting biomarker data.^6^

Limitations include not adjusting for body mass index, occupation, physical activity, hormonal status, and medication use, the cross-sectional study design, and pooling data from 2 study sites. Nevertheless, the results highlight low grip strength as a potential transdiagnostic biomarker, reflecting motor and motivational dysfunction, persisting into remission, and showing diagnosis and sex-specific symptom associations. This underscores the value of grip strength for early detection and intervention that should be examined in other psychiatric disorders beyond schizophrenia and depression.

## Notes

Supplement 1. eMethods eReferences

Supplement 2. Data sharing statement
