# Supplementary material for: Transdiagnostic Patterns of Grip Strength in Schizophrenia, Current Depression, and Remitted Depression
Source: JAMA Psychiatry. 2026 Mar 18;83(5):546–9. doi: 10.1001/jamapsychiatry.2026.0144 (PMC13000739; doi:10.1001/jamapsychiatry.2026.0144)
Supplement: Supplement 1. — eMethods eReferences [file jamapsychiatry-e260144-s001.pdf]

Supplemental Online Content

von Känel S, Pavlidou A, Nadesalingam N, et al. Transdiagnostic patterns of grip strength in schizophrenia, current, and remitted depression. *JAMA Psych*. Published online March 18, 2026. doi:10.1001/jamapsychiatry.2026.0144

eMethods.
eReferences.

This supplemental material has been provided by the authors to give readers additional information about their work.

© 2026 von Känel S et al. *JAMA Psych*.

eMethods. The data was derived from five different studies. Participants from these studies were included in this analysis if data were available for all variables investigated. Four studies were conducted in Bern, Switzerland (Overcoming Psychomotor Slowing in Psychosis (OCoPS-P)[1]; The Brain Stimulation and Group Therapy to Improve Gesture and Social Skills in Psychosis Trial (BrAGG-SoS)[2]; Accelerated Repetitive Transcranial Magnetic Stimulation for Psychomotor Slowing (ATMSSlowing), BASEC 2023-D0031, NCT06134661; Gestures in depression, BASEC 2023-00309) and one study was conducted in Chicago, USA (PSANDS)[3]. For the clinical trials (OCoPS-P, BrAGG-SoS, ATMSSlowing) only baseline data was included. Patients with schizophrenia were included from the OCoPS-P (n=83), BrAGG-SoS (n=81), and ATMSSlowing study (n=11). Patients with current depression were included from the Gestures in depression (n=29) and the PSANDS study (n=50), and patients with remitted depression from the PSANDS study (n=104). The healthy controls were from the OCoPS-P (n=22), BrAGG-SoS (n=37), Gestures in depression (n=27), and the PSANDS study (n=89). In sum, all patients with schizophrenia (100%), 29 patients with current depression (37%), and 86 control participants (49%) were from Bern, while 50 patients with current depression (63%), all participants with remitted depression (100%), and 89 healthy controls (51%) were from Chicago. Age in the schizophrenia group was significantly higher compared to the other groups (cMDD: z=3.6, p=0.001; rMDD: z=5.3, p<.001; HC: z=2.6, p=0.014) and higher in the control compared to the rMDD group (z=3.04, p=0.005). The current depression group showed no significant age differences compared to both the remitted depression group and the control group. Sex-distribution only differed between the schizophrenia and the current (X^2=8.5, p=0.018) and between schizophrenia and the remitted depression groups (X^2=7.6, p=0.018), with the current and remitted depression groups consisting of more female participants than the schizophrenia group.

Patients with schizophrenia and current depression were heterogenous groups with varying levels of symptom severity, recruited from both outpatient (schizophrenia n=71; current depression n=61) and inpatient (schizophrenia n=104; depression, n=18) facilities. Inpatient facilities provided acute psychiatric care, e.g. for patients experiencing an acute deterioration of their condition. None of the participants were long-term hospitalized.

Specific inclusion criteria for patients treated in the OCoPS-P and the ATMSSlowing study were having schizophrenia spectrum disorders with severe psychomotor slowing as measured with the Salpêtrière Retardation Rating Scale (SRRS). In addition, the OCoPS-P study also included clinical controls (24 out of 83 patients with schizophrenia participating in this study) without psychomotor slowing. Participants in the rMDD group were included if they had a past

© 2026 von Känel S et al. *JAMA Psych.*

but no current DSM-5 diagnosis for major depressive disorder and a MADRS score of seven or less. rMDD patients were excluded if they currently met any other major DSM diagnosis (i.e., anxiety, obsessive-compulsive disorder, or eating disorder) or criteria for moderate/severe alcohol and substance abuse. Since all studies involved magnetic resonance imaging (MRI), standard MRI exclusion criteria such as metal implants or pregnancy applied.

At the time of testing, 69.0% of patients were treated with antipsychotic medication (95.5% with schizophrenia (97% of inpatients and 94% of outpatients) and 10.1% with current depression (28% of inpatients and 5% of outpatients) and 41.6% with antidepressant medication (17.6% with schizophrenia (4% of inpatients and 35% of outpatients), 49.4% with current depression (83% of inpatients and 43% of outpatients), and 34.6% with remitted depression). For four participants (one with schizophrenia, three with current depression) information on medication was not available. In total 95% of the participants with schizophrenia and 100% of participants in the other groups were right-handed. All grip strength measures were conducted following a standardized protocol with participants sitting in an upright position. All participants across sites and studies used identical instruments (electronic dynamometer, model EH101, CAMRY, Hong Kong). Three participants (one patient with schizophrenia and two healthy controls) were excluded due to abnormal recordings related to technical issues.

Analyses were performed in R Studio (version 4.3.2). Age was compared between groups using Dunn's tests, while sex distribution was assessed with a chi squared test. Differences in grip strength between groups were analyzed using a linear regression model with sex and age included as covariates due to those variables differing between groups. In addition, due to the studentized Breusch–Pagan test[4] indicating heteroskedasticity (BP = 45.63, df = 5, $p < .001$), and residuals showing minor deviations from normality (Shapiro–Wilk $W = 0.99$, $p = .01$; $N = 533$), linear regression model estimates and post hoc comparisons were conducted with heteroskedasticity-robust (HC3) standard errors[5]. Because none of the participants in the healthy control and the remitted depression groups were inpatients, the effect of inpatient status on grip strength was assessed by conducting a sensitivity analysis restricted to patients with current depression and schizophrenia, where we included inpatient status alongside age and sex as a co-variate. Like our main regression model, partial Spearman's rank correlations investigating associations between grip strength and symptoms were controlled for age and sex. If interesting patterns emerged, we conducted separate sex-specific correlation analyses while controlling for age. We used the Benjamini-Hochberg method to control for multiple comparisons across 12 tests.

© 2026 von Känel S et al. *JAMA Psych*.

eReferences

1. Walther S, Alexaki D, Weiss F, et al. Psychomotor Slowing in Psychosis and Inhibitory Repetitive Transcranial Magnetic Stimulation. *Jama Psychiatry*. Jun 5 2024;doi:10.1001/jamapsychiatry.2024.0026
2. Walther S, Maderthaner L, Chapellier V, et al. Gesture deficits in psychosis and the combination of group psychotherapy and transcranial magnetic stimulation: A randomized clinical trial. *Mol Psychiatry*. Dec 2025;30(12):5790–5799. doi:10.1038/s41380-025-03303-7
3. Shankman SA, Mittal VA, Walther S. An Examination of Psychomotor Disturbance in Current and Remitted MDD: An RDoC Study. *Journal of Psychiatry and Brain Science*. 2020;5(2):e200007. e200007. doi:10.20900/jpbs.20200007
4. Breusch TS, Pagan AR. Simple Test for Heteroscedasticity and Random Coefficient Variation. *Econometrica*. 1979;47(5):1287–1294. doi:Doi 10.2307/1911963
5. Aslam M. Using Heteroscedasticity-Consistent Standard Errors for the Linear Regression Model with Correlated Regressors. *Commun Stat-Simul C*. Nov 26 2014;43(10):2353–2373. doi:10.1080/03610918.2012.750354

© 2026 von Känel S et al. *JAMA Psych*.
