# Supplementary material for: Transdiagnostic Patterns of Grip Strength in Schizophrenia, Current Depression, and Remitted Depression
Source: JAMA Psychiatry. 2026 Mar 18;83(5):546–9. doi: 10.1001/jamapsychiatry.2026.0144 (PMC13000739; doi:10.1001/jamapsychiatry.2026.0144)
Supplement: Supplement 2. — Data sharing statement [file jamapsychiatry-e260144-s002.pdf]

Data Sharing Statement

von Känel. Transdiagnostic Patterns of Grip Strength in Schizophrenia, Current Depression, and Remitted Depression. *JAMA Psychiatry*. Published March 18, 2026.
doi:10.1001/jamapsychiatry.2026.0144

Data
Data available: No

Additional Information
Explanation for why data not available: The participants did not consent to data sharing.
